# Lenvatinib Plus Paclitaxel as Second‐Line Therapy for Advanced Gastric Cancer Patients: A Dose Escalation Exploratory Study

**DOI:** 10.1002/advs.202506678

**Published:** 2025-08-11

**Authors:** Chenfei Zhou, Jinling Jiang, Liting Guo, Wenqi Xi, Junwei Wu, Qu Cai, Jun Ji, Feng Qi, Jun Zhang

**Affiliations:** ^1^ Department of Oncology Ruijin Hospital Shanghai Jiao Tong University School of Medicine Shanghai 200025 China; ^2^ Shanghai Institute of Digestive Surgery Shanghai Jiao Tong University School of Medicine Shanghai 200025 China; ^3^ Shanghai Key Laboratory of Gastric Neoplasms Shanghai 200025 China

**Keywords:** dose escalation, dynamic network biomarker, gastric cancer, lenvatinib, second‐line

## Abstract

Second‐line treatment options for advanced gastric cancer (AGC) patients remain limited. This dose escalation exploratory study is aimed to determine the maximum tolerated dose (MTD) of lenvatinib plus paclitaxel as second‐line treatment in AGC and to explore molecular biomarkers using dynamic network biomarker (DNB) analysis. Eligible patients are treated with lenvatinib plus paclitaxel (135 mg m^−2^, q3w). Dose escalation of lenvatinib adopts a “3 + 3” design. DNB analysis is performed based on Olink proteomics data using serial blood samples. Eleven patients are treated. No dose‐limiting toxicities are observed, and the MTD of lenvatinib is determined as 16 mg. White blood cells decreased (45.5%, 5/11) is the most common adverse event. Grade ≥3 adverse event rate is 18.2% (2/11). Objective response rate is 36.4% (4/11), and median overall survival is 7.4 months. A DNBscore associated with patient prognosis and response to multitarget tyrosine kinase inhibitor (TKI) plus paclitaxel is established. Infiltration of cancer‐associated fibroblasts in tumor microenvironment is positively associated with the DNBscore. In summary, lenvatinib plus paclitaxel is well tolerant and shows promising efficacy as a second‐line regimen in AGC. The DNBscore can be a biomarker for multitarget TKI plus paclitaxel and provide critical clues for future research .

## Graphical abstract

This study determines the maximum tolerated dose of lenvatinib in combination with paclitaxel as second‐line therapy for advanced gastric cancer patients. A dynamic network biomarker panel associated with patient prognosis and response to treatment is established. The finding provides information for patient enrichment and new target selection in future clinical trials of angiogenesis inhibitor‐based treatment in gastric cancer.

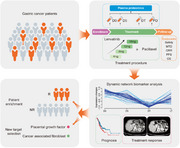

## 1 Introduction

Gastric cancer (GC) is one of the leading causes of cancer‐related mortality in China.^[^ ^1^ ^]^ Despite comprehensive treatment, the 5‐year survival rate of patients with advanced diseases remains unsatisfied.^[^ ^2^, ^3^ ^]^ Combination chemotherapy is the backbone of systemic treatment for advanced gastric cancer (AGC) patients.^[^ ^4^ ^]^ In the last decade, novel agents targeting critical molecules such as programmed cell death 1 (PD‐1), erb‐b2 receptor tyrosine kinase 2 (HER2), and Claudin‐18.2 have been approved for the first‐line treatment of AGC patients in combination with standard chemotherapy, which improves patients’ median overall survival to ≈16 months.^[^ ^5^, ^6^, ^7^ ^]^

Although significant advances have been achieved in first‐line treatment, available subsequent treatment options for AGC patients after disease progression are still limited. Paclitaxel or irinotecan is the standard second‐line chemotherapy regimen, while angiogenesis inhibitors in combination with chemotherapy have emerged as an effective strategy.^[^ ^4^ ^]^ Ramucirumab, a monoclonal antibody targeting vascular endothelial growth factor receptor 2 (VEGFR‐2), has been approved for previously treated gastric cancer patients.^[^ ^8^ ^]^ The oral tyrosine kinase inhibitor (TKI) fruquintinib targeting VEGFR 1–3 showed superior antitumor efficacy in combination with paclitaxel versus paclitaxel alone for second‐line treatment of gastric cancer.^[^ ^9^ ^]^ However, the survival improvement of patients remains modest, which underscores an urgent need to develop novel second‐line treatment options for gastric cancer patients.

Understanding the mechanisms of cancer resistance to treatment is important for developing novel antitumor strategies.^[^ ^10^ ^]^ In a previous study, we performed circulating tumor DNA (ctDNA) analysis using serial blood samples from AGC patients collected during first‐ and second‐line chemotherapy, and found that genomic alterations of ctDNA at disease progression of first‐line platinum‐based chemotherapy could be enriched into several cancer‐related signaling pathways but showed significant heterogeneity among patients. Furthermore, these alterations were barely influenced by conventional second‐line chemotherapy.^[^ ^11^ ^]^ These findings can help explain the poor efficacy of conventional chemotherapy as well as single‐target agents for second‐line treatment of gastric cancer.^[^ ^12^ ^]^ Based on these results, we hypothesized that in combination of a multitarget inhibitor could be a potential subsequent therapeutic for AGC patients.

Lenvatinib is a multitarget TKI targeting VEGFR 1–3, fibroblast growth factor receptors (FGFR) 1–4, platelet‐derived growth factor receptor β (PDGFR‐β), rearranged during transfection (RET), and receptor tyrosine kinase (KIT).^[^ ^13^ ^]^ Its potent efficacy in AGC has been shown in the EPOC1706 trial with an objective response rate (ORR) at 69% in 29 AGC patients treated with lenvatinib plus pembrolizumab. The multitarget merit of lenvatinib and its potent efficacy in gastric cancer based on available clinical evidence, indicate it can be a candidate for our hypothesis. However, the toxicities of lenvatinib at the commonly used dose should be noted. In the EPOC1706 trial, all patients experienced at least one dose reduction due to adverse events when treated with 20 mg of lenvatinib once daily.^[^ ^14^ ^]^ The combination of lenvatinib with chemotherapy as second‐line therapy for AGC patients has not yet been evaluated, and its optimal dose remains to be determined.

In the present study, we aimed to assess lenvatinib plus paclitaxel as second‐line therapy for HER2‐negative AGC patients. The primary objective was to characterize the safety profile and determine the maximum tolerated dose (MTD) of lenvatinib in this regimen. The preliminary treatment efficacy was also observed. Plasma proteomics data of serial blood samples collected during treatment were analyzed to explore potential molecular biomarkers.

## 2 Results

### 2.1 Clinical Characteristics of Patients

**Figure 1:**
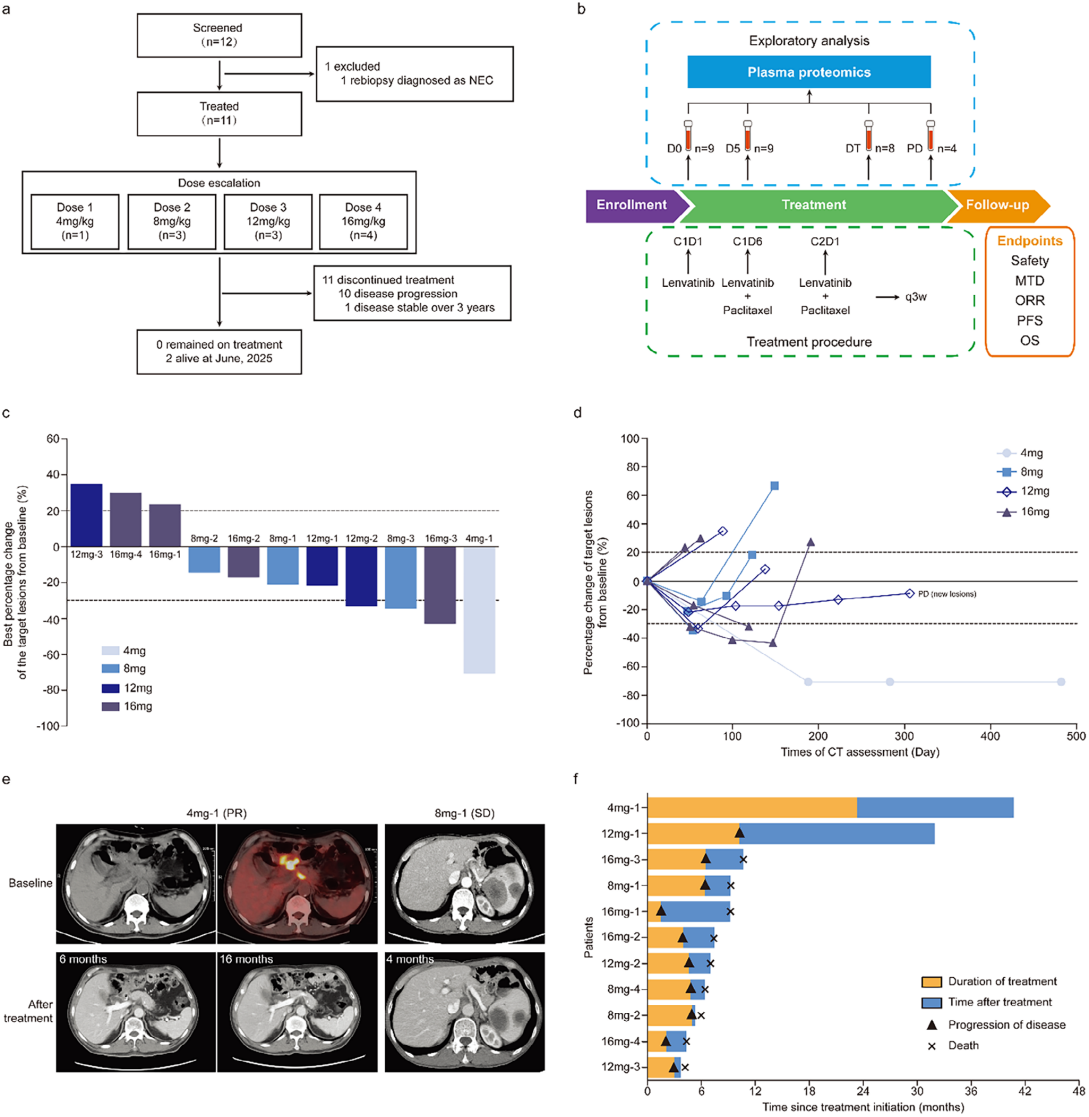
Study profiles and treatment outcomes of patients. a) CONSORT diagram. HER2 negative AGC patients were screened and enrolled into 4 dose groups (*n* = 11). As of June 2025, 2 patients remained alive. The main reason for discontinued treatment was disease progression. NEC: neuroendocrine carcinoma. b) The study procedure was illustrated. Peripheral blood samples were collected, including 9 samples at baseline (D0), 9 samples at 5 days after lenvatinib treatment (D5), 8 samples at radiological assessment during treatment (DT), and 4 samples at disease progression (PD). c) The best percentage change of target lesions from baseline; dotted lines at 20% and −30% indicated progressive disease and partial response, respectively. d) Dynamic change of target lesions from baseline during treatment. e) Representative CT images of patients before and after treatment. f) Duration of treatment and survival of patients.

From January 2022 to October 2023, 11 patients were enrolled (**Figure** **1**a). There were 8 males and 3 females with the median age at 63 years (range, 35–71). All patients received platinum plus fluoropyrimidines as first‐line treatment, and 3 of them received PD‐1 antibody (**Table** **1**). A total of 30 peripheral blood samples were collected from patients at baseline (9 cases, D0), day 5 after lenvatinib treatment (9 cases, D5), during treatment (8 cases, DT), and progressive disease (4 cases, PD, Figure 1b)

**Table 1:** Clinicopathological characteristics of patients.

Clinicopathological characteristics	N [*n* = 11]	Percentage [%]
Median age, years (range)	63 (35–71)	/
Gender		
Male	8	72.7
Female	3	27.3
ECOG		
0	2	18.2
1	9	81.8
Primary lesion		
Resected	8	72.7
Un‐resected	3	27.3
Number of metastatic organs		
1	8	72.7
≥2	3	27.3
Peritoneal metastasis		
Yes	4	36.4
No	7	63.6
First‐line treatment		
SOX/XELOX	7	63.6
Capecitabine plus cisplatin	1	9.1
SOX + Sintilimab	1	9.1
FOLFOX + Sintilimab	1	9.1
XELOX + Sintilimab	1	9.1

FOLFOX: oxaliplatin, leucovorin calcium and fluorouracil; SOX: S‐1 plus oxaliplatin; XELOX: Capecitabine plus oxaliplatin;

### 2.2 Safety

All patients were assessable for safety analysis. No dose‐limiting toxicity (DLT) was observed in all dose groups. MTD of lenvatinib was determined as 16 mg. Treatment‐emergent adverse events (TEAEs) of any grade occurred in 9 patients (81.8%, **Table** **2**). White blood cell count decreased was the most common adverse event (5/11, 45.5%). Grade ≥3 TEAEs were reported by 2 patients, both in the 16 mg dose group, including hypertension (1, 9.1%), increased blood bilirubin (1, 9.1%), and biliary tract infection (1, 9.1%). No dose reduction of lenvatinib was reported. Treatment delay of paclitaxel was reported by 1 patient (12 mg group) due to grade 2 platelet count decreased.

**Table 2:** Treatment‐emergent adverse events (TEAEs) of patients.

TEAEs [*n* = 11]	All [%]	G1 [%]	G2 [%]	G3/4 [%]
Any	9 (81.8)	7 (63.6)	3 (27.3)	2 (18.2)
Neutrophil count decreased	3 (27.3)	2 (18.2)	1 (9.1)	
White blood cell count decreased	5 (45.5)	4 (36.4)	1 (9.1)	
Platelet count decreased	3 (27.3)	2 (18.2)	1 (9.1)	
Hypertension	2 (18.2)	1 (9.1)		1 (9.1)
Blood bilirubin increased	2 (18.2)	1 (9.1)		1 (9.1)
AST/ALT increased	2 (18.2)	2 (18.2)		
Peripheral sensory neuropathy	2 (18.2)	2 (18.2)		
Anemia	1 (9.1)	1 (9.1)		
Hypoalbuminemia	1 (9.1)		1 (9.1)	
Hyperkalemia	1 (9.1)	1 (9.1)		
Urinary tract infection	1 (9.1)		1 (9.1)	
Biliary tract infection	1 (9.1)			1 (9.1)

ALT: alanine transaminase; AST: aspartate transaminase; G: grade.

### 2.3 Efficacy

Treatment efficacy was assessed in 11 patients (Table S1, Supporting Information). No complete response (CR) was achieved, and partial response (PR) was observed in 1 patient of each dose group (Figure 1c). ORR was 36.4% (4/11), and disease control rate (DCR) was 72.7% (8/11). Change of target lesions during treatment was illustrated in Figure 1d. The representative CT images of disease‐controlled patients are shown in Figure 1e. As of June 2025, the median follow‐up time was 7.4 months, and 2 patients were still alive. Median progression‐free survival (PFS) and overall survival (OS) were 4.8 months (95%CI, 3.7 to 5.9) and 7.4 months (95%CI, 4.4 to 10.5). The duration of response of the patient in 4 mg group exceeded 40 months (Figure 1f).

### 2.4 Identification of Efficacy‐Related Dynamic Network Biomarkers (DNBs)

**Figure 2:**
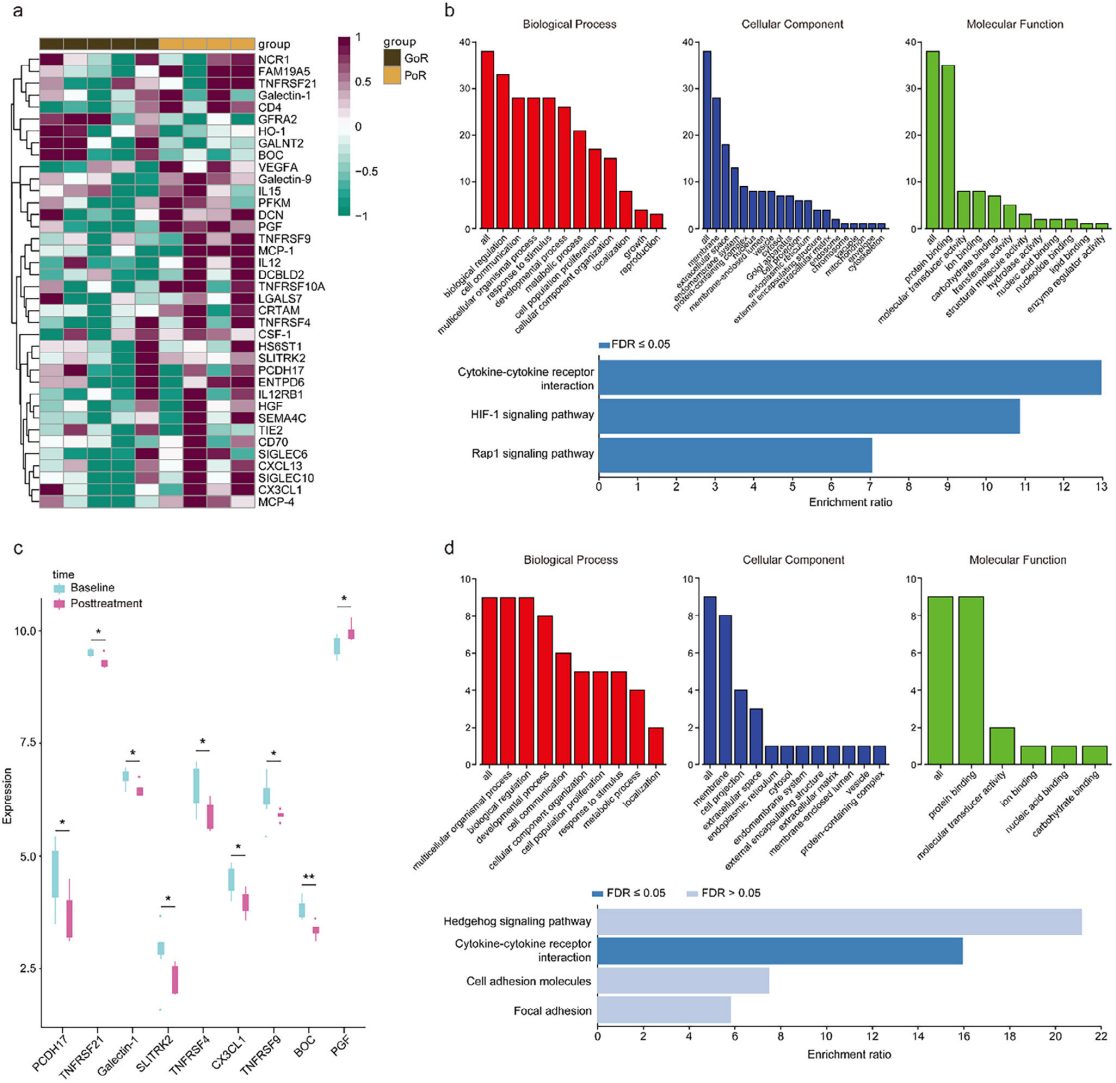
Identification of efficacy‐related DNBs. a): Heatmap of DNBs in D0 samples of GoR (*n* = 5) and PoR (*n* = 4) groups. b) GO analysis and KEGG pathway analysis of DNBs in D0 samples. c) Relative expression of DNBs at baseline and posttreatment (*n* = 9; ^*^ *p* < 0.05; ^**^ *p* < 0.01). d) GO analysis and KEGG pathway analysis of differential DNBs after treatment.

By using partial least squares‐discriminant analysis (PLS‐DA) and DNB analysis, levels of 38 proteins were found different between good responders (GoRs) and poor responders (PoRs) in D0 samples and all samples, and were identified as DNBs (Figure S1, Table S2, Supportive Information). At baseline, the relative levels of most DNBs were higher in PoRs (**Figure** **2**a). KEGG pathway analysis showed that these proteins could be enriched into cytokine‐cytokine receptor interaction, HIF‐1 signaling pathway, and Rap1 signaling pathway (Figure 2b; Table S3, Supportive Information). Comparing the plasma levels of DNBs before (D0 samples) to after treatment (LT samples), 9 proteins were found to be different. Among them, the level of plasma placental growth factor (PGF) was found to be higher in PoRs and was increased after treatment (Figure 2c). KEGG pathway analysis based on 9 proteins showed cytokine–cytokine receptor interaction was also enriched (Figure 2d, Table S4, Supportive Information).

### 2.5 A DNB Scoring System Associated with the Prognosis of Gastric Cancer Patients

**Figure 3:**
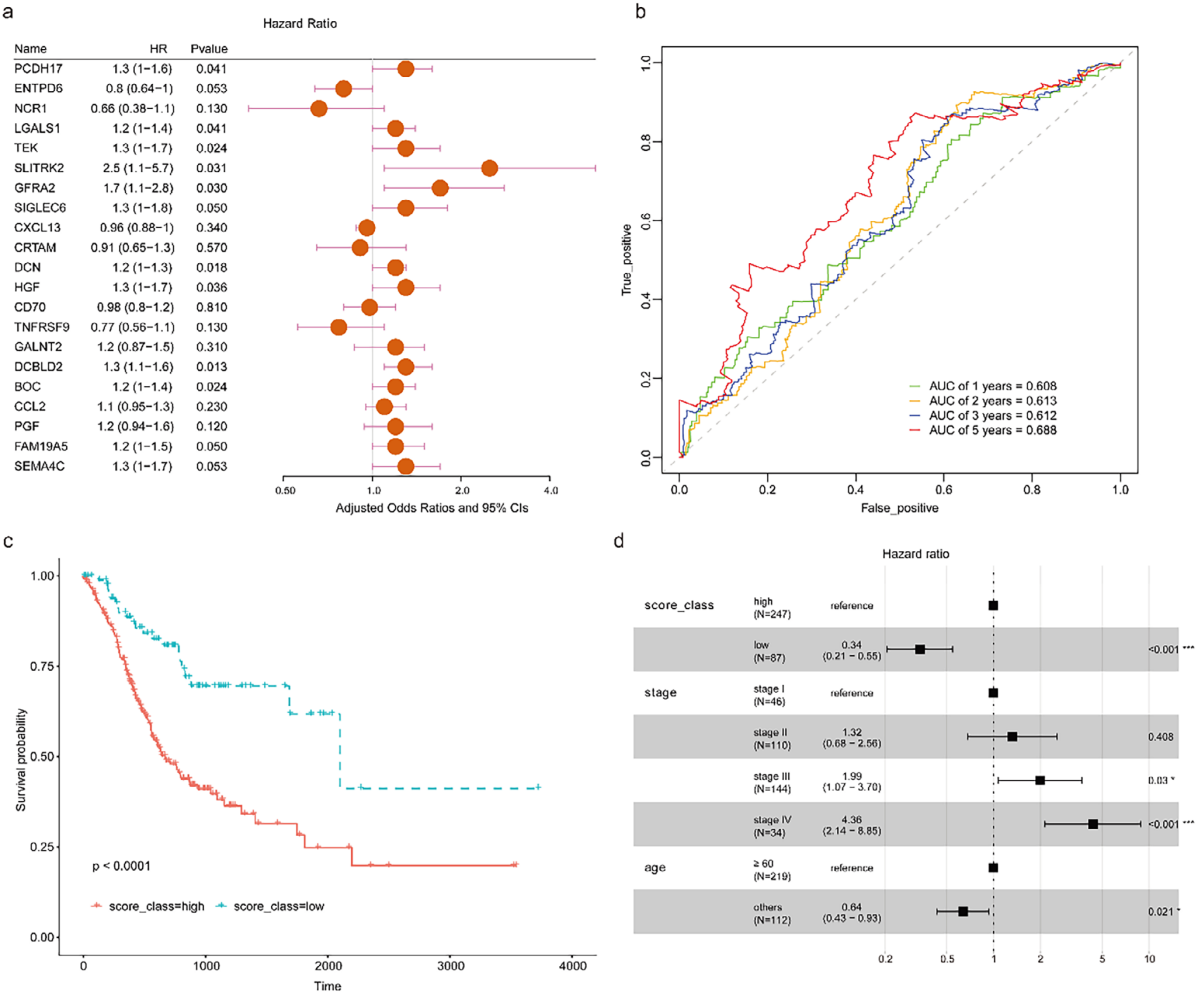
Construction of the DNB scoring system. a) Prognosis analysis of DNBs in the TCGA‐STAD cohort. b) Receiver operating characteristic curve analysis of DNBscore for patients’ survival in the TCGA‐STAD cohort. c) The median value of DNBscore was used to stratify patients into DNBscore‐low and ‐high groups. Kaplan–Meier curves of DNBscore‐high and DNBscore‐low patients in the TCGA‐STAD cohort were illustrated. Overall survival between the 2 groups showed a significant difference (*p*<0.0001). d) Forest plot of multivariate Cox regression analysis showed that the DNBscore, disease stage, and age were the independent prognostic factors in the TCGA‐STAD cohort.

To investigate the functional role of DNBs in gastric cancer, gene expression of all 38 proteins was analyzed in the TCGA‐STAD cohort. Differential expression of 22 genes was found between normal and tumor samples (Figure S2, Supportive Information). Prognosis analysis showed that 21 genes were associated with patients’ survival (**Figure** **3**a). A DNB scoring system (DNBscore) was then established based on the expression value and prognostic coefficient of 21 genes in gastric cancer samples (Figure 3b; Table S5, Supportive Information). Patients with high DNB scores (above the median value) showed worse survival than those with low DNB scores (Figure 3c). Multivariate Cox proportional hazard models revealed that the DNBscore was an independent prognostic factor for gastric cancer patients (Figure 3d).

### 2.6 The DNBscore Indicated the Response to Multitarget TKI Plus Paclitaxel in Gastric Cancer

**Figure 4:**
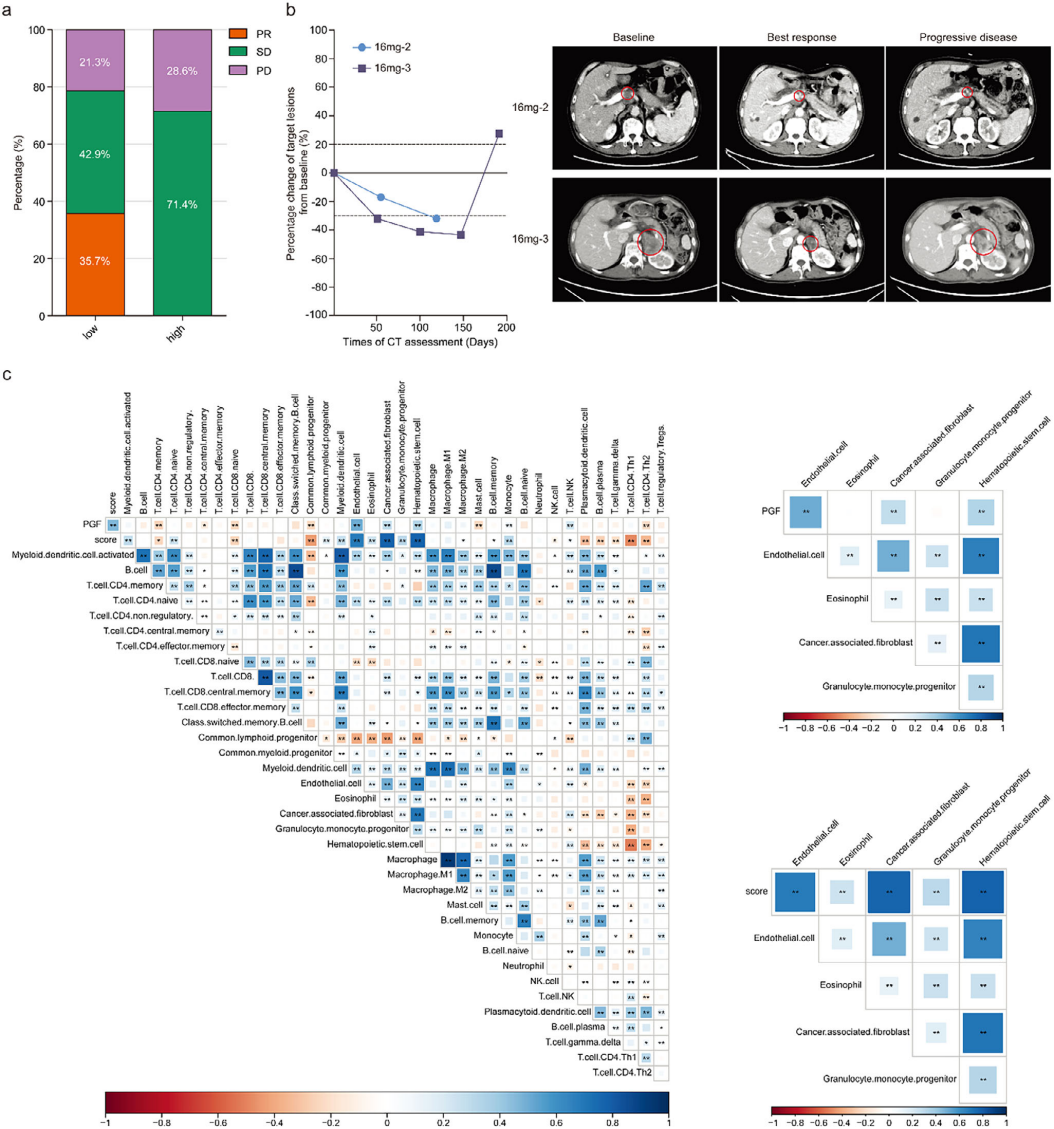
Association of DNBscore with treatment response and cell infiltration in TME. a) Treatment response of DNBscore‐low (*n* = 14) and ‐high (*n* = 7) patients treated with regorafenib plus paclitaxel. b) Dynamic change of target lesions from baseline during treatment of 2 patients determined as DNBscore‐low in our study. Representative CT images of patients were illustrated. c) Correlation analysis of DNBscore and PGF with stromal cells in tumor microenvironment (^*^ *p* < 0.05; ^**^ *p* < 0.01).

To analyze the association between DNBscore and efficacy of multitarget TKI‐based second‐line therapy in gastric cancer, RNA sequencing data from 21 esophagogastric carcinoma patients treated with regorafenib and paclitaxel as second‐line therapy in REPEAT study were used (GSE198136), and DNBscore was calculated. PR, stable disease (SD), and PD were achieved in 5 (23.8%), 11 (52.4%), and 5 (23.8%) patients, respectively. Among them, 14 patients were DNBscore‐low and PR rate was 35.7% (5/14). No PR was observed in the DNBscore‐high group (0%, 0/7, **Figure** **4**a). The 2 patients in our study were both determined as DNBscore‐low (Table S6, Supportive Information). The male patient (16mg–2) who had a metastatic retroperitoneal lymph node as the target lesion experienced tumor shrinkage over 30%, while he stopped study treatment due to obstructive jaundice. The female patient (16mg–3) achieved PR, and the duration of response was 6.5 months (Figure 4b).

### 2.7 The DNBscore and Cell Infiltration Phenotype in Tumor Microenvironment

Differentially expressed genes (DEGs) were analyzed between DNBscore‐low and ‐high groups using the GSE198136 dataset (Figure S3, Supportive Information). Fibroblast‐related genes, including *COL1A2*, *COL1A1*, *COL3A1*, *COL8A1*, and *COL6A3* were identified as low‐expressed DEGs in the DNBscore‐low group. The association between DNBscore and stromal cells in the tumor microenvironment (TME) was analyzed. DNBscore was positively associated with infiltration of cancer‐associated fibroblast (CAF), hematopoietic stem cell, and endothelial, while was negatively associated with immune cells, including CD4^+^Th1 cell, CD4^+^Th2 cell, CD8^+^ naïve T cell, and NK cell. PGF, as a key efficacy‐related protein, was also positively associated with infiltration of CAF (Figure 4c).

## 3 Discussion

In this study, the MTD of lenvatinib in combination with paclitaxel as a second‐line regimen for AGC patients was determined. A DNB scoring system was established that was associated with patient prognosis and response to multitarget TKI plus paclitaxel therapy. The DNBscore was also associated with an unfavorable TME phenotype of gastric cancer.

Anti‐angiogenesis is one of the effective strategies for AGC patients after first‐line therapy. Lenvatinib has been approved to treat multiple tumors, including renal cell carcinoma (RCC), unresectable hepatocellular carcinoma (HCC), differentiated thyroid cancer (DTC), and endometrial carcinoma (EC).^[^ ^15^, ^16^, ^17^, ^18^, ^19^ ^]^ Given the success of ramucirumab and fruquintinib in AGC, lenvatinib in combination with chemotherapy is worthy to be investigated due to its multitarget merit. However, the recommended doses of lenvatinib vary across indications, and the frequency of grade ≥3 adverse events (AEs) and dose reduction is high. In the EPOC1706 trial, AEs led to dose interruption in 97% patients and dose reduction of lenvatinib in 100% gastric cancer patients.^[^ ^14^ ^]^ In RCC and DTC, more than 60% of patients initiating on 24 mg daily required at least one dose reduction, and the final doses (median 20.3 mg day^−1^ in RCC and mean 17.2 mg day^−1^ in DTC) were lower than the starting dose across both populations.^[^ ^15^, ^17^ ^]^ When combined with pembrolizumab in EC and RCC at 20 mg daily, the AE‐related dose reduction rate of lenvatinib also exceeded 60%.^[^ ^18^, ^19^ ^]^ In contrast, body weight‐based dosing of lenvatinib in HCC resulted in a comparatively lower AE‐related dose reduction rate of 37%.^[^ ^16^ ^]^

In this study, lenvatinib at 16 mg once daily was determined as MTD for AGC patients in combination with paclitaxel (135 mg m^−2^, every 3 weeks). Hematologic toxicities were the common AEs, and lenvatinib‐related grade ≥3 AEs, including diarrhea, proteinuria, and palmar‐plantar erythrodysesthesia, were not observed. The MTD of lenvatinib plus weekly paclitaxel (80 mg m^−2^) in advanced endometrial and ovarian cancer was also determined as 16 mg, but 60% of patients initiated at or below MTD still required a dose reduction of lenvatinib.^[^ ^20^ ^]^ A relatively low intensity of paclitaxel administrated in our study might result in the low incidence of grade ≥3 AEs. Lenvatinib, administrated at either 4 mg twice daily or 8 mg once daily in combination with chemotherapy, has been evaluated at first‐line in non‐small cell lung cancer or advanced intrahepatic cholangiocarcinoma.^[^ ^21^, ^22^ ^]^ However, the LEAP‐015 trial showed that lenvatinib at 8 mg once daily plus pembrolizumab and chemotherapy for AGC patients resulted in grade ≥3 AEs in 79% of participants which was higher than chemotherapy alone, and 24% of participants experienced lenvatinib discontinuation.^[^ ^23^ ^]^ The different safety profiles of lenvatinib in various combination regimens underscore the necessity of indication‐specific dose optimization of lenvatinib. Based on our study, lenvatinib at 12 mg daily plus standard dose of docetaxel or weekly paclitaxel can be assessed in a future phase 2 trial.

Given the limited sample size, the efficacy of this regimen cannot be fully evaluated in our study. As second‐line regimens of AGC patients, regorafenib or fruquintinib plus chemotherapy have been assessed in randomized trials. In the RAINBOW trial, the ORR in the ramucirumab plus paclitaxel group was 28% and 16% in the paclitaxel group, with superior PFS (4.4 months vs 2.9 months, *p*<0.001) and OS (9.6 months vs 7.4 months, *p* = 0.017) in the combination group.^[^ ^8^ ^]^ A higher ORR in fruquintinib plus paclitaxel group compared with paclitaxel alone (42.5% vs 22.4%, *p* < 0.0001) was also observed in the FRUTIGA trial, with a superior PFS (5.6 months vs 2.7 months, *p* < 0.0001).^[^ ^9^ ^]^ In our study, the doses of lenvatinib and paclitaxel are relatively low due to the dose escalation design and the uncertain toxicity profile. Notably, meaningful clinical activity can be observed, with tumor regression and long‐term survival achieved even at low dose groups.

The LEAP‐015 trial demonstrated significant improvements in PFS and ORR in the lenvatinib combination group versus chemotherapy alone in AGC. However, the prespecified threshold for OS significance was not met, and the ORR in the lenvatinib group (59.5%) was also relative lower than that reported in the EPOC1706 trial (69%).^[^ ^23^ ^]^ In the era of precision medicine, the conventional “one‐size‐fits‐all” approach has been proven clinically ineffective.^[^ ^24^ ^]^ These results underscore the critical need for biomarker‐guided enrollment in lenvatinib‐based clinical trials, while no validated biomarker for lenvatinib has yet been established in gastric cancer. Therefore, we performed an exploratory DNB analysis, which is a method enabling time‐resolved identification of efficacy‐related molecular signatures under systemic therapy. Unlike conventional comparisons between samples at baseline and disease progression, DNB analysis can identify the molecules exhibiting substantial fluctuations during treatment, which suggests significant response to systemic therapy and involvement in treatment response or resistance mechanisms.^[^ ^25^, ^26^ ^]^

As a result, an efficacy‐related DNB panel for this regimen is identified. By integrating RNA sequencing data, associations between molecules in the DNB panel and patient prognosis are found, which indicate their functional involvement in tumor progression. Furthermore, the DNBscore can stratify patient prognosis and indicate treatment response to multitarget TKI plus paclitaxel in gastric cancer. Currently, few studies have focused on molecular biomarker analysis for second‐line treatment in gastric cancer. Our finding suggests that the DNBscore can be a potential tool to enrich gastric cancer patients who are likely to benefit from multitarget TKI‐based treatment. Due to the inherent limitation of the dose‐escalation study design, definitive validation of the predictive value of the DNBscore cannot be achieved. Nevertheless, these findings will inform our planned phase 2 or randomized controlled trial with appropriate power to validate the predictive value of the DNBscore.

The association between the DNB score and unfavorable TME phenotype should be noted. Specifically, DNBscore showed a positive correlation with infiltration of CAF in TME. Although the resistance mechanism of lenvatinib in gastric cancer remains unclear, previous studies in HCC have demonstrated that CAF activation is strongly associated with lenvatinib resistance.^[^ ^27^ ^]^ CAF‐derived secreted phosphoprotein 1 activates bypass signaling pathways in HCC cells, conferring resistance to TKI‐induced apoptosis and promoting tumor progression.^[^ ^28^ ^]^ PGF is identified as a key protein associated with the treatment efficacy of lenvatinib plus paclitaxel in the DNB panel. A positive association between PGF expression and CAF infiltration is also found. PGF belongs to the vascular endothelial growth factor (VEGF) family, binding to VEGFR1, and can be produced by CAF.^[^ ^29^, ^30^ ^]^ Its high expression is detected in various tumors and correlated with pathological angiogenesis.^[^ ^31^ ^]^ Elevated plasma PGF was detected in AGC patients who had a poor response to ramucirumab.^[^ ^32^ ^]^ Kim, et al. reported that chemotherapy induced expression of PGF in pancreatic cancer, which directly activated CAFs and led to treatment failure. A novel VEGF decoy receptor targeting PD‐L1‐expression CAFs via the PD‐L1 directed PGF/VEGF blockade could improve the efficacy of chemotherapy.^[^ ^33^ ^]^ In phase I trials, long‐term disease control was achieved by some patients with advanced solid tumors who were treated by PGF monoclonal antibody.^[^ ^34^, ^35^ ^]^ Therefore, targeting PGF can be a promising strategy for gastric cancer patients with high DNB score or PGF level and should be validated in our future studies.

There were several limitations of the present study. First, the sample size was small. Dose expansion phase was not designed in this trial due to the uncertain toxicity and rapid progression of novel agents in gastric cancer. Based on current results, we recommend lenvatinib at 12 mg combined with a standard dose of chemotherapy to be evaluated in a phase 2 study. Furthermore, the integration of DNBscore as a biomarker and targeting PGF can be options for future clinical trial design. Second, paired tumor tissue samples were not collected for exploratory analysis. Re‐biopsy in AGC patients is usually difficult due to procedure‐related risks. In this study, liquid biopsy was used as an alternative strategy, and public RNA sequencing datasets were used. Given the limited availability of large‐scale datasets for second‐line treatment of gastric cancer, further validation of the clinical utility of the DNBscore is warranted.

In conclusion, lenvatinib plus paclitaxel is well‐tolerated and shows promising efficacy as a second‐line regimen for AGC patients. The DNBscore can be a potential tool to guide patient enrollment, while PGF is a promising molecular target in subsequent treatment of gastric cancer.

## 4 Experimental Section

### Study Design and Participants

This was a dose escalation exploratory study conducted at the Department of Oncology, Ruijin Hospital, Shanghai. The study was performed in accordance with the Declaration of Helsinki and the guidelines of Good Clinical Practice. The protocol was approved by the Ethics Committee of Ruijin Hospital, Shanghai Jiao Tong University School of Medicine, Shanghai, People's Republic of China (2021, No.290) and was registered on Clinicaltrial.gov website (NCT05171530, registered on 29 December 2021). Written informed consent was provided by all patients before screening.

Eligible patients were aged over 18 years and below 80 years, HER2‐negative and pathological confirmed gastric or gastro‐esophageal junction adenocarcinoma with locally advanced unresectable or metastatic diseases, treatment failure after first‐line regimens, with at least one radiologically evaluable lesion according to Response Evaluation Criteria in Solid Tumor (RECIST, version 1.1); Eastern Cooperative Oncology Group (ECOG) performance status of 0 or 1 with life expectancy over 3 months and adequate organ function (white blood cell count ≥3.0 × 10^9^/L; neutrophil count ≥1.5 × 10^9^/L; hemoglobin ≥85 g L^−1^; PLT ≥100 × 10^12^/L; total bilirubin ≤1.5×upper normal limit (ULN); alanine aminotransferase (ALT) and aspartate aminotransferase (AST) ≤2.5 × ULN and ≤5 × ULN for patients with liver metastases; blood creatinine ≤1.5 × ULN; proteinuria less than 2+). Key exclusion criteria included previous exposure to lenvatinib or taxane, with pregnancy or lactation, symptomatic metastases of the central nervous system, other primary malignancies, history of active gastrointestinal bleeding within 3 months, bowel obstruction or other conditions affecting oral administration, and uncontrollable blood pressure or other comorbidities.

### Procedures

Eligible patients received lenvatinib orally once daily and intravenous paclitaxel at a dose of 135 mg per square meter every 3 weeks until disease progression, intolerance to toxicity, or withdrawal of consent. At the first cycle, lenvatinib monotherapy was given for 5 days ahead to monitor safety, then paclitaxel was administered on day 6. For dose escalation of lenvatinib, a modified 3 + 3 design was adopted. Four dose levels were set, including 4, 8, 12, and 16 mg. One patient was enrolled in the 4 mg dose group as an accelerated titration. If no DLT was observed, a standard 3 + 3 protocol was performed.

DLTs were observed within the 21‐day period of the first cycle and were defined as grade ≥4 hematological toxicities; grade 3 white blood cell count decreased, or platelet count decreased, which could not be recovered after 1 week of treatment; grade ≥4 non‐hematological toxicities, expect controllable high blood pressure and abnormal laboratory examination. TEAEs were recorded and assessed at the first day of every cycle according to the Common Terminology Criteria for Adverse Events (CTCAE, version 5.0). Treatment was temperately suspended if grade ≥3 toxicities occurred. Patients were treated following clinical protocol until recovery, then the dose of paclitaxel was reduced by 20% and lenvatinib was reduced by one dose level. Treatment was discontinued permanently if grade ≥3 toxicities occurred again after dose reduction.

Demographic information of patients was recorded at baseline. Physical examinations, routine hematological tests, coagulation function, thyroid function, urine test, fecal occult blood test, and tumor marker test were performed every 3 weeks on day 1 of each cycle. Tumor response was assessed by radiological examination (contrast‐enhanced computed tomography preferred) according to RECIST criteria by investigators at baseline and every 9 weeks until disease progression. Peripheral blood samples of each patient were collected at baseline (D0), 5 days after lenvatinib administration in the first cycle (D5), radiological examination (during treatment, DT), and progressive disease (PD) for the following exploratory analysis. Samples collected at progressive disease or the last samples of patients who didn't have disease progression were assigned as the last time of sampling (LT) for exploratory analysis.

### Endpoints

The primary endpoint was to determine MTD of lenvatinib based on DLTs. Safety profiles, including TEAEs and severe adverse events (SAEs), were recorded. The secondary endpoints included ORR, DCR, PFS, and OS. ORR was defined as the proportion of patients with a best objective response of CR or PR. DCR was defined as the proportion of patients with the best objective response of CR, PR, or SD. CR was defined as the disappearance of all target lesions. PR was defined as at least a 30% decrease in the sum of diameters of target lesions. PD was defined as at least a 20% increase in the sum of diameters of target lesions or the appearance of new lesions. SD was defined as neither sufficient shrinkage to qualify for PR nor sufficient increase to qualify for PD. PFS was defined as the time from enrollment to first documented progressive disease or death, whichever occurred first. OS was defined as the time from enrollment until the data of death from any cause. The exploratory endpoint was to investigate the proteomic data in peripheral blood samples during treatment and to explore potential biomarkers. Patients achieved CR, PR, or SD (≥4 months) were stratified as GoRs, and patients with SD (<4 months) and PD were stratified as PoRs for exploratory analysis.

### Proteomics Analysis

One microliter of plasma from each sample was used as input material for detection. Proximity Extension Assay in the Olink platform was performed based on a matched pair of antibodies linked to unique oligonucleotides binding to the respective protein target, and the DNA amplicon was subsequently quantified by quantitative real‐time PCR. Two panels were used (Olink Target 96 Immune‐Oncology panel, 95320, and Cell Regulation panel, 95370. File S1, Supporting Information). In the incubation phase, the antibody pairs labeled with DNA oligonucleotides bound to the respective protein in the samples. In the extension and amplification phase, oligonucleotides were brought into proximity to hybridize, and were extended using a DNA polymerase. The DNA barcodes were then amplified by PCR. Finally, the amount of each DNA barcode was quantified by microfluidic qPCR. The microfluidic qPCR was quantified by Olink Signature Q100, and the data were read out by Olink normalized protein expression (NPX) Signature software. The incubation, extension, and detection of the Olink experiment were performed by Sinotech Genomics Co., Ltd (Shanghai, China). NPX of each protein was calculated from threshold cycles, and data pre‐processing (normalization) was performed to minimize both intra‐ and inter‐assay variation. Individual protein levels across the sample set were determined by NPX data (Files S2 and S3, Supporting Information).

### Partial Least Squares‐Discriminant Analysis (PLS‐DA)

PLS‐DA model was utilized for multivariate statistical analysis based on Olink proteomic data via the “Ropls” R package to identify differential proteins between GoRs and PoRs in D0 samples and all samples. As a supervised discriminant analysis method, PLS‐DA maximizes inter‐group differences based on predefined classification variables and achieves superior sample separation compared to principal component analysis. Variable importance in projection (VIP) scores were calculated to evaluate the contribution of each protein to the classification. Proteins with VIP≥1 were selected as potential biomarkers for subsequent validation. A total of 69 and 78 proteins were identified between GoRs and PoRs in D0 samples and all samples, respectively.

### Dynamic Network Biomarker (DNB) Analysis

Dynamic changes analysis via the “Mfuzz” R package on the Olink proteomic data was performed to get gene clusters and their changes among each sampling time point (D0, D5, DT, and LT). DNBs were identified according to the nonlinear dynamic theory. Four indices of each cluster were calculated, including average standard division of DNBs, average Pearson correlation coefficient among DNBs (PPC*i*), average Pearson correlation coefficients between DNBs and non‐DNBs (PPC*o*), and criticality index (CI). The DNBs should meet the following criteria: 1) the expression of DNBs fluctuates widely during treatment; 2) PPC*i* among DNBs at protein levels were changed significantly; 3) PPC*o* between DNBs and non‐DNBs are decreased significantly. Following steps are performed to identify DNBs during treatment by using D0 samples as reference: 1) calculate standard division of each protein at each sampling time point, and choose genes whose standard division in each time point at least 2 times more than standard division in D0; 2) cluster the selected genes at each time point; 3) normalize the level of each protein at each time point; 4) select cluster that match the criteria of DNB and have a highest CI.^[^ ^26^ ^]^ A total of 49 proteins were identified. The proteins in DNB panel were selected as the overlap between DNB analysis and PLS‐DA analysis.

### Establishment of the DNBscore

The expression of identified genes in the DNB panel between normal and tumor tissues was compared using the transcriptomics sequencing data and clinical data of the TCGA‐STAD cohort collected from the UCSC Xena (https://xenabrowser.net/). Single‐factor Cox regression analysis was performed to analyze the association between each identified gene and patient survival in the TCGA‐STAD cohort. Expression value of gene (Exp gene) and β value (the independent prognostic coefficient) calculated based on single‐factor Cox regression of each gene were recorded, and were used to establish the DNBscore according to the following formula: score = ∑ β genes × Exp genes. The median value of DNBscore was used to stratify patients into DNBscore‐low and ‐high groups, and the Kaplan‐Meier method was performed to compare the survival of the two groups.

### DNBscore and Clinical Phenotype of Gastric Cancer

The transcriptomics sequencing data and clinical data of gastric cancer patients treated with regorafenib and paclitaxel as second‐line therapy were collected from the NCBI dataset (GSE198136).^[^ ^36^ ^]^ Batch effects were removed using the “ComBat” function in the package “sva” with TCGA‐STAD samples as the reference for batch adjustment. Patients were stratified into DNBscore‐low and ‐high groups calculated by the DNB scoring system. Cut‐off value of DNBscore associated with objective response was determined by the receiver operating characteristic curve. Differentially expressed genes (DEGs) between the DNBscore‐low and ‐high groups were compared using transcriptomics sequencing data. RNA sequencing data of 2 samples collected from patients enrolled in our study were used to calculate the DNBscore. An association between DNB‐score and cell infiltration in the tumor microenvironment of gastric cancer was performed based on the online tool TIMER2.0 (http://timer.cistrome.org/) and CIBERSORT algorithm (https://cibersort.stanford.edu/).

### Statistical Analysis

The sample size for the dose escalation trial was determined by safety profiles during study practices, and a minimum of 6 subjects was required. Clinical characteristics, safety data, and efficacy data, including ORR and DCR, were summarized by frequency and percentage. Differential expression analysis for protein was performed using the Olink Analyze R package (version 3.1.0). Comparisons of continuous variables were tested by Student's *t*‐test, with non‐normally distributed data tested by the Mann‐Whitney test. Two‐sided *P* values less than 0.05 were considered statistically significant (level of significance: *p < 0.05; **p < 0.01; ns, *p* > 0.05). Survival curves were constructed and tested using the Kaplan–Meier method and the log‐rank test. The association between the clinical information and DNBscore groups was examined using the chi‐square test. Univariate and multivariate Cox proportional hazard regression models adjusted or not adjusted for available prognostic clinical covariates, were performed to calculate hazard ratios and 95% confidence intervals. Correlation analysis was conducted with Spearman's correlation. GO analysis for biological processes, cellular components, and molecular function, and KEGG pathway analysis of differential proteins were performed via WEB‐based GEne SeT AnaLysis (webgestalt). All statistical analyses were performed with R software (version 3.6.3) for statistical computing or GraphPad Prism software (version 8.0).

### Ethics Approval Statement

All procedures conducted in studies involving human participants were aligned with the ethical standards of the institutional and/or national research committee, as well as with the Helsinki Declaration and its subsequent amendments. The protocol was approved by the Ethics Committee of Ruijin Hospital, Shanghai Jiao Tong University School of Medicine, Shanghai, People's Republic of China (2021, No.290) and were registered on Clinicaltrial.gov website (NCT05171530).

## Conflict of Interest

The authors declare no conflict of interest.

## Author Contributions

C.Z., J.J., and L.G. contributed equally to this work. C.Z. and J.Z. designed the research. C.Z., J.J., L.G., W.X., J.W., and F.Q. conducted the clinical trial, including patient enrollment, treatment, assessment, and follow‐up. Q.C. and J.J. contributed to the clinical sample collection and processing. F. Q. performed the bioinformatic analysis. F. Q. and C.Z. analyzed and visualized the data. C.Z. wrote the original draft, and all authors revised and approved the final manuscript. J.J. supervised the project and provided funding.

## Supporting information



Supporting Information

Supplementary Table 1

Supplementary Table 2

Supplementary Table 3

Supplementary Table 4

Supplementary Table 5

Supplementary Table 6

Supplementary File 1

Supplementary File 2

Supplementary File 3

## Data Availability

The data that support the findings of this study are available from the corresponding author upon reasonable request.
