# Supplementary material for: Lenvatinib Plus Paclitaxel as Second‐Line Therapy for Advanced Gastric Cancer Patients: A Dose Escalation Exploratory Study
Source: Adv Sci (Weinh). 2025 Aug 11;12(40):e06678. doi: 10.1002/advs.202506678 (PMC12561184; doi:10.1002/advs.202506678)
Supplement: Supplementary file 1 — Supporting Information [file ADVS-12-e06678-s008.docx]

**Supporting information**


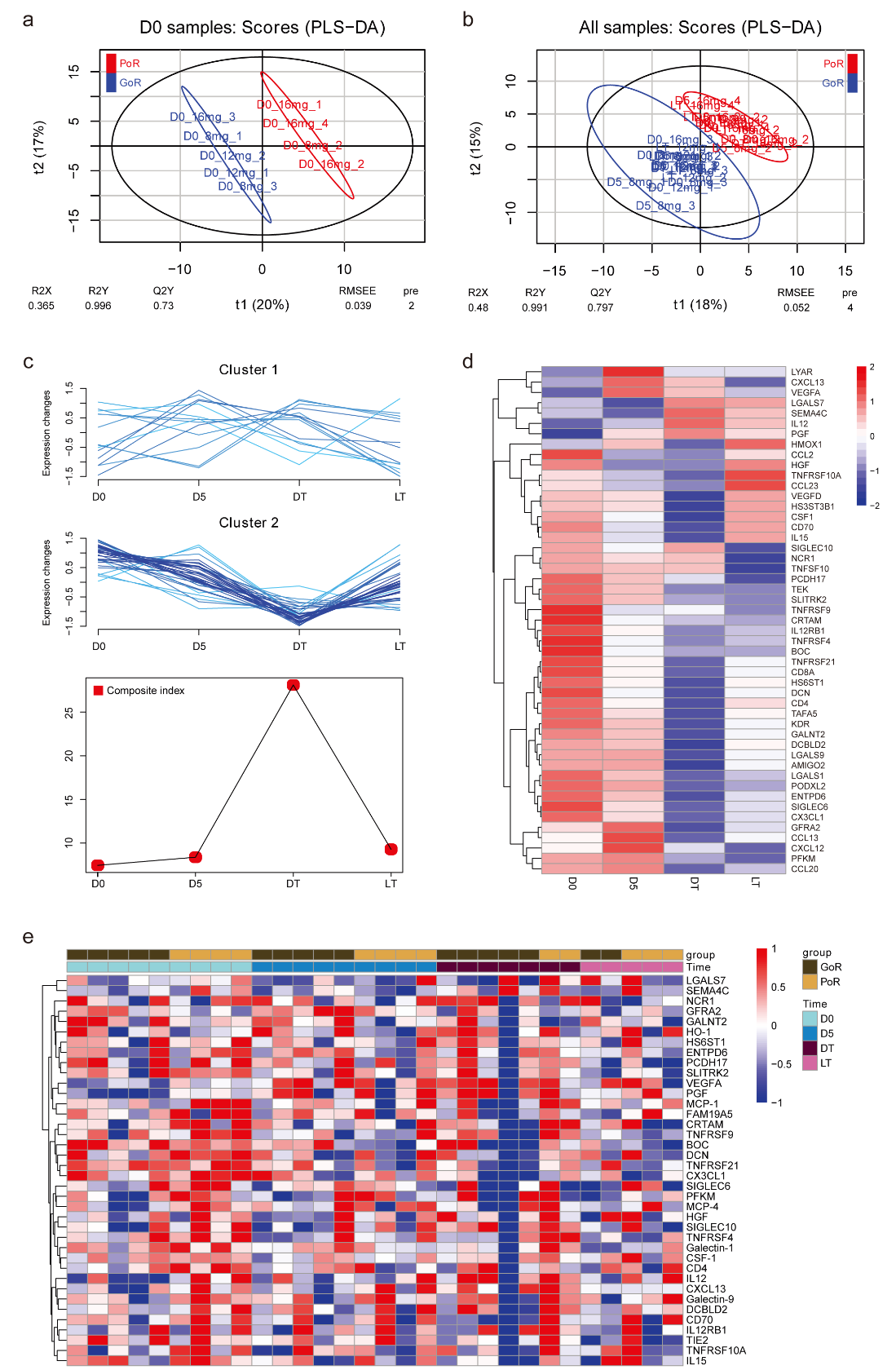
**Title:** Lenvatinib plus paclitaxel as second-line therapy for advanced gastric cancer patients: a dose escalation exploratory study

**Figure S1.** PLS-DA and DNB analysis. a) PLS-DA analysis of D0 samples between GoR and PoR groups. b) PLS-DA analysis of all samples between GoR and PoR groups. c) Dynamic changes of relative expression of proteins and composite index during treatment. d) Heatmap of DNBs relative expression during treatment. e) Heatmap of DNB panel in all samples.


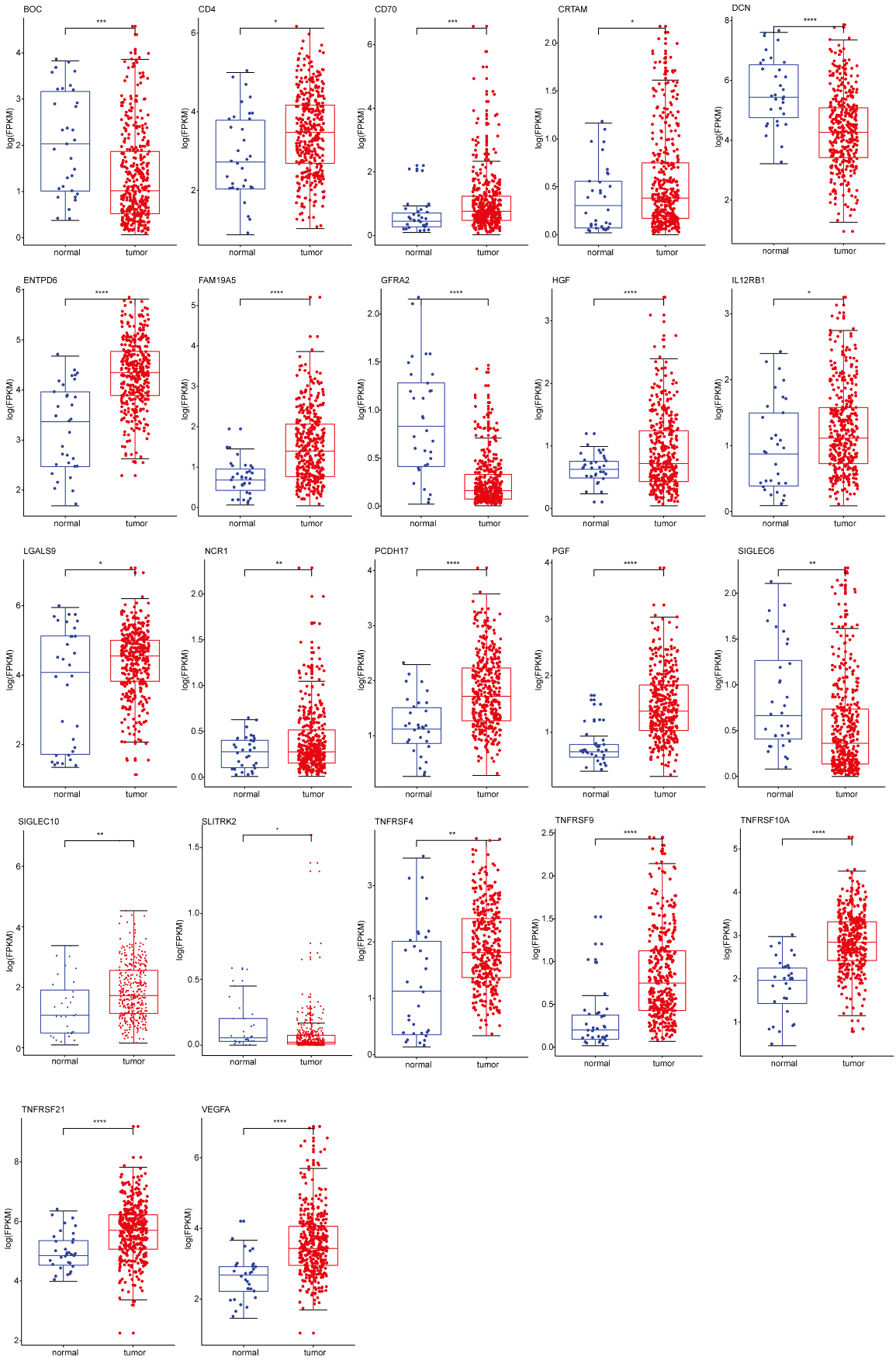


**Figure S2.** Relative expression of DNBs between normal and tumor samples in the TCGA-STAD cohort. Expression of 22 genes in the DNB panel were found different between normal and tumor samples in the TCGA-STAD cohort (**P* < 0.05; ***P* < 0.01; ****P* < 0.001).


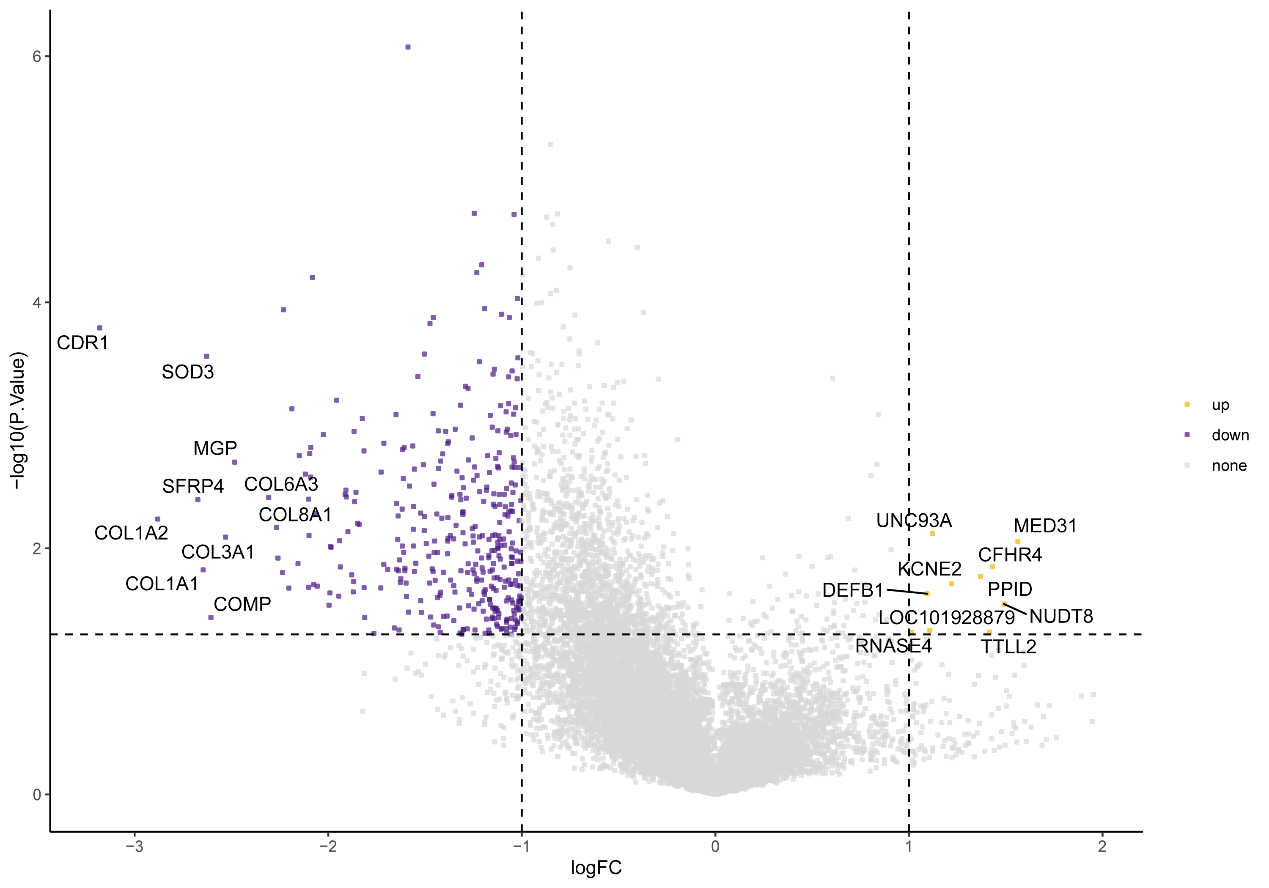


**Figure S3.** Differential expressed genes in DNBscore-low group compared with -high group

**Table S1** Patients’ tumor burden and treatment efficacy

**Table S2** List of DNBs

**Table S3** KEGG analysis of 38 DNBs

**Table S4** KEGG analysis of 9 DNBs

**Table S5** Single-factor Cox regression of 21 DNBs for DNB scoring system establishment

**Table S6** Expression of 21 genes in DNB panel and DNBscore of samples

**File S1** Olink target panel

**File S2** NPX data of protein in Cell Regulation panel
**File S3** NPX data of protein in Immune-Oncology panel
