# Supplementary material for: Lenvatinib Plus Paclitaxel as Second‐Line Therapy for Advanced Gastric Cancer Patients: A Dose Escalation Exploratory Study
Source: Adv Sci (Weinh). 2025 Aug 11;12(40):e06678. doi: 10.1002/advs.202506678 (PMC12561184; doi:10.1002/advs.202506678)
Supplement: Supplementary file 2 — Supplementary Table 1 [file ADVS-12-e06678-s002.docx]

**Table S1** **Patients’ tumor burden and treatment efficacy**

| Dose level | Case | Gender | Age (years) | Metastatic sites | Size of target lesions  (mm) | Response | PFS (months) | OS (months) |
| --- | --- | --- | --- | --- | --- | --- | --- | --- |
| 4mg | 1 | M | 71 | Lymph nodes | 17 | PR | 40.77 | 40.77 |
| 8mg | 2 | M | 56 | Spleen, liver, lymph nodes | 104 | SD | 6.37 | 9.23 |
|  | 3 | M | 67 | Liver, lung, lymph nodes | 42 | SD | 4.97 | 5.30 |
|  | 4 | F | 55 | Liver | 37.3 | PR | 4.80 | 6.40 |
| 12mg | 5 | M | 69 | Lung | 23 | SD | 10.23 | 31.93 |
|  | 6 | F | 35 | Peritoneum | 12 | PR | 4.60 | 7.00 |
|  | 7 | M | 68 | Lymph nodes | 63 | PD | 2.97 | 3.73 |
| 16mg | 8 | M | 68 | Peritoneum | 34 | PD | 1.50 | 9.20 |
|  | 9 | M | 60 | Lymph nodes | 20 | SD | 3.97 | 7.43 |
|  | 10 | F | 63 | Lymph nodes, adrenal gland | 67 | PR | 6.47 | 10.63 |
|  | 11 | M | 40 | Peritoneum, lymph nodes | 20 | PD | 2.10 | 4.33 |

F: female; M: male; OS: overall survival; PD: progressive disease; PFS: progression-free survival; PR: partial response; SD: stable disease;
